# Nationwide analysis of laparoscopic groin hernia repair in Italy from 2015 to 2020

**DOI:** 10.1007/s13304-022-01374-7

**Published:** 2022-09-07

**Authors:** Monica Ortenzi, Emanuele Botteri, Andrea Balla, Mauro Podda, Mario Guerrieri, Alberto Sartori

**Affiliations:** 1 Department of General Surgery, Università Politecnica Delle Marche, Piazza Roma 22, 60121 Ancona, Italy; 2 General Surgery, ASST Spedali Civili Di Brescia PO Montichiari, Via Boccalera 325018, Montichiari, Brescia, Italy; 3 UOC of General and Minimally Invasive Surgery, Hospital “San Paolo”, Largo Donatori del Sangue 1, 00053 Civitavecchia, Rome, Italy; 4 Department of Surgical Science, Emergency Surgery Unit, University of Cagliari, Cagliari, Italy; 5 Department of General Surgery, Ospedale Di Montebelluna, Via Palmiro Togliatti, 16, 31044 Montebelluna, Treviso, Italy

**Keywords:** Laparoscopy, TEP, TAPP, Groin hernia, Nationwide analysis

## Abstract

Since its introduction, the minimally invasive treatment of groin hernias has become widely accepted as a viable alternative to open surgery. Still, the rates and reasons for its adoption vary highly among countries and the regions within a country. After almost thirty years since its introduction, its spread is still limited. The present study, conducted under the auspices of AGENAS (Italian National Agency for Regional Services), aims at giving a snapshot of the spreading of minimally invasive and robotic techniques for the treatment of groin hernia in Italy. This study is retrospective, with data covering the period from 1st January 2015 to 31st December 2020. AGENAS provided data using the operation and diagnosis codes used at discharge and reported in the International Classification of Diseases 9th revision (ICD9 2002 version). Admissions performed on an outpatient basis, i.e., without an overnight stay of at least one night in hospital, were excluded. A total of 33,925 laparoscopic hernia repairs were performed during the considered period. Overall, a slight increase in the number of procedures performed was observed from 2015 to 2019, with a mean annual change of 8.60% (CI: 6.46–10.74; *p* < 0.0001). The number of laparoscopic procedures dropped in 2020, and when considering the whole period, the mean annual change was − 0.98% (CI: − 7.41–5.45; *p* < 0.0001). Urgent procedures ranged from 335 in 2015 to 508 in 2020 referring to absolute frequencies, and from 0.87% to 9.8% in relative frequencies of overall procedures in 2017 and 2020, respectively (mean = 4.51%; CI = 3.02%–6%; *p* < 0.001). The most relevant observation that could be made according to our analysis was that the adoption of the laparoscopic approach knew a slow but steady increase from 2015 onward.

**Supplementary Information:**

The online version contains supplementary material available at 10.1007/s13304-022-01374-7.

## Introduction

The surgical risks and technical difficulties initially hindered the spread of the minimally invasive approach to groin hernia. The possible severe complications and the need for general anesthesia to treat a benign disease that could instead be treated with little risk and under local anesthesia through the open anterior approach contributed to the slowdown in the spread of minimally invasive techniques. After an initial difficulty, several studies and subsequently the EHS (European Hernia Society) and EAES (European Association of Endoscopic Surgery) guidelines have demonstrated the safety and the advantages of the laparoendoscopic approach in the treatment of groin hernia [1, 2]. The high incidence of the disease has made groin hernia repair the most widely performed surgery today, with about 20 million operations per year. About 1.6 million visits are made each year in the United States for problems related to inguinal-crural hernias; the lifetime risk of developing an inguinal hernia is approximately 27–43% in males and 3–6% in females [3, 4]. The initial indications for treating inguinal hernias by laparoendoscopic approach were recurrences after the anterior approach and bilateral inguinal hernias, thus reducing the scope of this approach [5]. However, the Hernia Surge Group has recently shown that the laparoendoscopic approach can be considered safe even for unilateral inguinal hernias when performed by experienced surgeons [1]. The present study, conducted under the auspices of AGENAS (Italian National Agency for Regional Services), aims at giving a snapshot of the spreading of minimally invasive and robotic techniques for the treatment of groin hernia in Italy.

## Materials and methods

This study is retrospective, with data covering the period from 1^st^ January 2015 to 31^st^ December 2020. AGENAS provided data using the operation and diagnosis codes used at discharge and reported in the International Classification of Diseases 9th revision (ICD9 2002 version). Admissions performed on an outpatient basis, i.e., without an overnight stay of at least one night in hospital, were excluded. Operations performed by laparoscopic and robotic techniques in patients older than 18 were considered. The coding and diagnosis codes are summarized in Table 1. Operations performed in association with minimally invasive surgery codes are also present in Table 1. Data from admission codes allowed for assessing gender, age, length of hospital stay and associated neurological and cardiovascular comorbidities. In addition, complications, readmission and 30-day mortality were assessed. No data were reported regarding the type of facility (public or private) where the operations were performed.

**Table 1:** Diagnosis and procedures coding system based on ICD-9-CM codes contained as primary interbentions/diagnosis or among the first five secondary intervention/diagnosis used to search for groin hernia data from 2015 to 2020 (source AgeNas)

	ICD-9-CM diagnosis code	ICD-9-CM treatment code
Monolateral inguinal hernia	550.00; 550.01; 550.02; 550.10; 550.11; 550.90; 550.91	53.00; 53.01; 53.02; 53.03; 53.04; 53.05
Bilateral inguinal hernia	550.00; 550;01; 550.02; 550.10; 550.11; 550.90; 550.91	53.10; 53.11; 53.12; 53.13; 53.14; 53.15; 53.16; 53.17
Monolateral femral hernia	551.00; 551.01; 552.00; 552.01; 553.00; 553.01	53.21; 53.29
Bilateral femoral hernia	552.02; 552.03; 553.03	53.31
Bowel obstruction	55.18; 5528; 55.29	
*Comorbidities*		
General comorbidities	25.00x (diabetis); 427.31 (atrial fibrillation); 585.9x (kidney failure); 491.20 (respiratory failure); 2865x-V5861 (anticoagulant)	
Neurological comorbidities	33.2xx (Parkinson); 29.00xx-29.03x (dementia); 331.0 (Alzheimer)	
Complications	998.11 (bleeding); 998.12 (hematoma); 998.12 (serohematoma); 99.60x-99.5x (infection) ‘AND’ 998.58–99.89x (wound) OR 996.87 (bowel)	
Associated procedures (AND)		
Cholecystectomy		51.23
Adhesiolisis		5451

### Statistical analysis

Data were processed using the MedCal statistical package (version 12.5). Qualitative variables were summarized by frequency and percentage, while normally distributed quantitative variables were described by the mean and standard deviation (SD). Statistical analysis was performed using Student's *t*-test and the Cochran Armitage test for trend as appropriate. A two-tailed *p*-value < 0.05 was considered statistically significant. The annual intervention rate (AIR) per 100,000 population was calculated, assessing the changes in the considered period. The sample size was the Italian population, reported by region, according to the average yearly population on 31st December from 2015 to 2020, reported by the Italian National Institute of Statistics (ISTAT) (Supplemental Table S1).

## Results

**Fig. 1:**
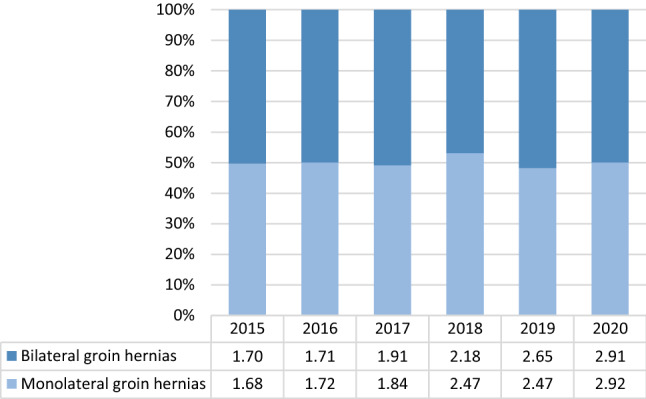
Monolateral and bilateral laparoscopic hernia repairs in absolute and relative frequencies performed in the index period (source AGENAS)

A total of 33,925 laparoscopic hernia repairs were performed during the considered period. Overall, a slight increase in the number of procedures performed was observed from 2015 to 2019, with a mean annual change of 8.60% (CI: 6.46–10.74; *p* < 0.0001). The number of laparoscopic procedures dropped in 2020, and when considering the whole period, the mean annual change was − 0.98% (CI: − 7.41–5.45; *p* < 0.0001). The percentage of laparoscopic procedures on the count of total procedures rose from 3.56% in 2015 to 5.98% in 2020. The percentage of laparoscopic procedures performed for bilateral inguinal hernias was almost similar to those performed for monolateral hernias in the whole period (Fig. 1).

The majority of patients were male (> 87% in the whole period), and the mean age was not statistically different (*p* = 0.972).

**Fig. 2:**
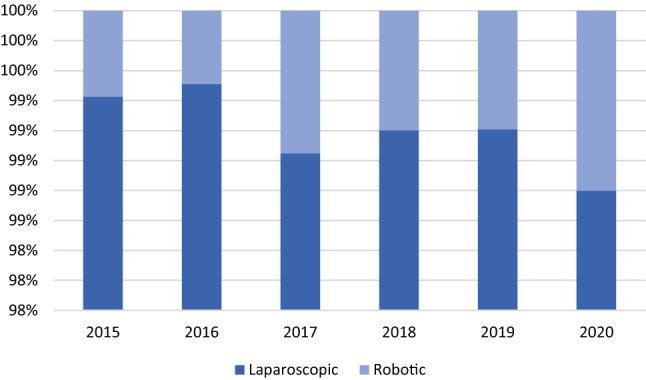
Laparoscopic and robotic hernia repairs in absolute and relative frequencies performed in the index period (source AGENAS)

The procedures performed with robotic assistance were 275 in total; however, the use of the robot increased in the considered period with a mean annual change of 10.67% (CI = 2.83%–18.51%) (Fig. 2).

The conversion rate to open surgery decreased from 2015 to 2019 with a mean annual change of − 1.14% (CI: − 10.2%–7.92%; *p* = 0.429). However, the decrease was not significantly different (*p* = 0.429) even when including 2020 in the analysis (*p* = 0.563).

**Fig. 3:**
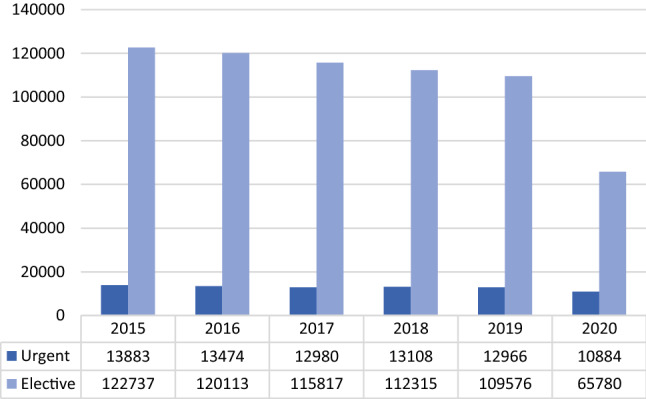
Elective and urgent procedures in absolute frequencies (source AGENAS)

Urgent procedures ranged from 335 in 2015 to 508 in 2020 referring to absolute frequencies, and from 0.87% to 9.8% in relative frequencies of overall procedures in 2017 and 2020 respectively (mean = 4.51%; CI = 3.02% – 6%; *p* < 0.001) (Fig. 3*).*

**Fig. 4:**
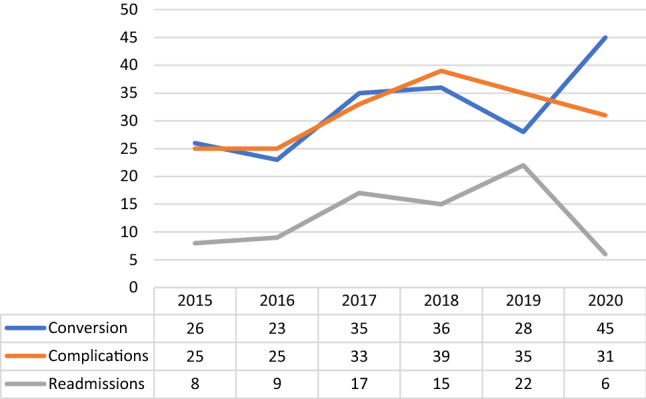
Conversion, Complication and readmission rate within 30 days rates from operation (source AGENAS)

Overall, there was a slight but not significant increase in the complication rate in the whole period (mean annual change = 3.06%; CI = − 1.94%–8%; *p* = 0.603). Conversely, in 2020, the readmission rate dropped, with a mean annual change of − 38% when considering the whole period (CI:− 77.16%–1.16%; *p* = 0.740), and an increase limited to the period from 2015 to 2019 (mean annual change = 19.16%; CI = − 10.33%–27.99%; *p* = 0.080) (Fig. 4).

**Fig. 5:**
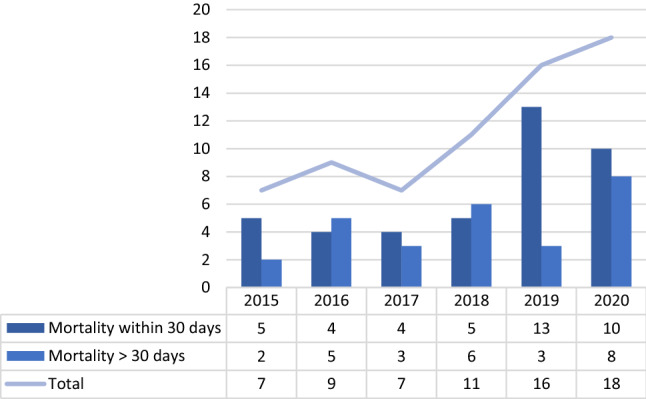
Early and late mortality rates (source AGENAS)

The overall mortality rate increased significantly when considering the whole period (mean annual change = 13.549%; CI = 4.82%–22.28%; *p* = 0.018), but this trend was not observed from 2015 to 2019 (mean annual change = 8.04%; CI = − 2.16%–18.24%; *p* = 0.280) (Fig. 5).

### Regional data

The number of elective procedures performed laparoscopically steadily increased all over Italy. However, the difference was insignificant in six regions, considering the whole period and the first five years without analyzing the 2020 data (Valle d'Aosta, Trentino, Veneto, Umbria, Molise, Campania and Sicily). Molise and Campania (AIR = 0) observed the minimum annual intervention rate, while the maximum was registered in Trentino (61 in 2019) Table 2.

**Table 2:** Regional data for laparoscopic elective procedures in the index period

	*Year*					
	2015	2016	2017	2018	2019	2020
*A. Annual Interventions Rate (Air) For urgent laparoscopic groin hernia procedures (100,000 Inhabitants) In Italy from 2015 to 2020 (Sources Agenas And Italian National Institute Of Statistics (2022) Resident Population On 31st December. ISTAT. *http://dati.istat.it/?lang=en#.)						
PIEMONTE	5	6	7	9	13	9
VALLE D'AOSTA	4	1	1	4	4	2
LOMBARDIA	14	14	13	15	18	10
TRENTINO ALTO ADIGE	50	54	53	53	61	47
VENETO	17	16	18	20	21	16
FRIULI VENEZIA GIULIA	14	16	13	22	21	20
LIGURIA	5	6	5	7	7	4
EMILIA-ROMAGNA	12	12	12	14	16	12
TOSCANA	7	8	10	11	14	10
UMBRIA	8	12	14	15	15	9
MARCHE	2	3	7	10	11	10
LAZIO	3	4	4	5	6	4
ABRUZZO	1	2	1	1	3	3
MOLISE	1	0	0	0	0	1
CAMPANIA	3	1	2	2	3	2
PUGLIA	3	3	4	3	6	4
BASILICATA	2	2	3	2	1	2
CALABRIA	1	1	0	0	1	1
SICILIA	3	4	3	4	4	4
SARDEGNA	3	2	4	4	5	9

	*Year*							
	2015	2016	2017	2018	2019	2020	*p*	*p*^1^
*B. Absolute numbers and for elective laparoscopic groin hernia procedures by region in Italy from 2015 to 2020 (sources Agenas)*								
Piemonte	219	253	305	369	540	378	< 0.0001	< 0.0001
Valle d'Aosta	5	1	1	5	5	2	0.788	0.860
Lombardia	1373	1352	1333	1548	1822	1003	< 0.0001	0.493
Trentino Alto Adige	529	574	567	571	663	509	0.102	0.122
Veneto	816	758	894	981	1040	797	0.530	0.757
Friuli Venezia Giulia	167	192	162	267	252	243	0.0003	0.034
Liguria	79	96	79	108	105	57	0.047	0.9243
Emilia-Romagna	512	530	531	628	710	549	0.048	0.034
Toscana	273	308	369	393	518	358	< 0.0001	< 0.0001
Umbria	74	106	125	131	133	82	0.942	0.069
Marche	34	53	101	147	167	144	< 0.0001	< 0.0001
Lazio	192	215	218	261	318	255	0.001	0.010
Abruzzo	15	20	14	14	36	42	< 0.0001	0.124
Molise	3	0	0	0	1	2	0.704	0.109
Campania	153	78	106	115	153	108	0.164	0.153
Puglia	106	127	171	133	221	157	0.001	0.001
Basilicata	12	12	15	10	7	10	0.119	0.060
Calabria	21	21	8	5	16	18	0.116	0.002
Sicilia	158	186	170	177	209	209	0.255	0.395
Sardegna	50	38	64	66	85	146	< 0.0001	0.027

*p*^1^ Cochrane Ermitage test without considering 2020

Concerning urgent procedures, an increase in the adoption of laparoscopy was observed. However, in 9 regions, this increase was not significant, considering the whole period and the first five years without analyzing the 2020 data (Valle d'Aosta, Trentino, Veneto, Liguria, Umbria, Abruzzo, Molise, Basilicata and Calabria). Furthermore, many regions showed the same annual intervention rate (AIR = 0), while the maximum was registered in Trentino (3).

Table 3 summarizes the distribution of urgent procedures in the index period.

**Table 3:** Regional data for laparoscopic urgent procedures in the index period

	*Year*					
	2015	2016	2017	2018	2019	2020
*A Annual Interventions Rate (AIR) for urgent laparoscopic groin hernia procedures (100,000 inhabitants) in Italy from 2015 to 2020 (sources Agenas and Italian National Institute of Statistics (2022) Resident population on 31st December. ISTAT. *http://dati.istat.it/?lang=en#*.)*						
Piemonte	0	0	0	1	1	1
Valle d'Aosta	2	0	0	3	2	1
Lombardia	1	1	1	1	1	1
Trentino Alto Adige	2	2	3	2	3	2
Veneto	1	1	1	1	1	1
Friuli Venezia Giulia	0	1	0	2	2	1
Liguria	1	1	1	1	1	1
Emilia-Romagna	1	1	1	1	1	1
Toscana	1	1	1	2	2	1
Umbria	1	1	1	1	1	1
Marche	0	0	0	1	1	1
Lazio	0	0	0	0	0	0
Abruzzo	0	0	0	0	0	0
Molise	0	0	0	0	0	0
Campania	0	0	0	0	0	0
Puglia	0	0	0	1	1	1
Basilicata	0	0	1	1	0	0
Calabria	0	0	0	0	0	0
Sicilia	0	0	0	0	0	0
Sardegna	0	0	1	1	1	1

	*Year*							
	2015	2016	2017	2018	2019	2020	*p*	*p*^*1*^
*B. Absolute numbers and for urgent laparoscopic groin hernia procedures by region in Italy from 2015 to 2020 (sources agenas)*								
PIEMONTE	18	19	17	22	27	33	0.022	0.223
VALLE D'AOSTA	3	0	0	4	2	1	0.926	0.691
LOMBARDIA	78	68	66	92	96	108	*0.005*	0.097
TRENTINO ALTO ADIGE	19	23	28	24	28	23	0.666	0.342
VENETO	52	42	41	48	48	64	0.339	0.640
FRIULI VENEZIA GIULIA	6	9	5	19	19	14	0.008	0.001
LIGURIA	9	14	11	8	9	11	0.597	0.442
EMILIA-ROMAGNA	38	40	35	43	53	53	0.080	0.204
TOSCANA	30	25	46	60	58	40	0.011	0.0001
UMBRIA	5	6	12	9	7	7	0.815	0.522
MARCHE	1	5	7	12	9	14	0.001	0.008
LAZIO	28	26	16	21	15	23	0.095	0.018
ABRUZZO	2	6	4	6	4	3	0.925	0.633
MOLISE	1	0	0	1	1	0	0.826	0.715
CAMPANIA	10	6	10	5	21	23	0.001	0.061
PUGLIA	9	15	13	25	44	49	< 0.0001	< 0.0001
BASILICATA	2	1	4	6	1	2	0.958	0.622
CALABRIA	3	2	1	1	2	2	0.591	0.437
SICILIA	8	15	12	17	20	18	0.060	0.051
SARDEGNA	7	4	11	13	12	14	0.039	0.073

*p*^1^ Cochrane Ermitage test without considering 2020

## Discussion

The present study provides an epidemiological snapshot of the laparoendoscopic treatment of groin hernias in Italy for the very first. The snapshot was obtained by processing the ICD9 Codes, and therefore the study provides a partial picture of the situation, although it is relatively indicative of the issue in Italy. The introduction in 1996 of the reimbursement system for medical procedures was developed to measure the productivity and intensity of work in hospital systems and was a real revolution in healthcare [6]. The data show a progressive increase in the laparoscopic approach to inguinal hernia over the last six years in all regions, although it is more significant in Northern Italy. The increasing trend was confirmed in 2020, although it was burdened by the COV-2 SARS pandemic afflicting the entire world. Globally, the minimally invasive approach is more widespread in wealthy countries, reaching high percentages in countries such as Australia (55%) and Switzerland (40%), probably determined both by the habits of surgeons and the welfare of the local health system [7, 8].

Nevertheless, an interesting observation is that despite the dramatic drop in the surgical caseload for benign disease in 2020, [9, 10] the rate of minimally invasive procedures across the total number of procedures performed raised to 5.98% in 2020, all groin hernia repairs performed.

Additionally, the increase in robotic procedures exceeded 2% of that observed for laparoscopic procedures. We explained the first observation as the surgery results in a few specialized centers with surgeons with the proper expertise in these procedures, whereas other less specialized centers abandoned groin hernia repair during the pandemic or referred the patients to other more qualified hospitals. [10]

As for robotic surgery, we think these data reflect the increasing robotic sprout we are witnessing in every surgical field. Future papers will show if this is connected to actual clinical benefits. [11]

Initially, the minimally invasive approach for treating inguinal hernias was hindered by the technical difficulties and a long learning curve associated with an operation that could be performed anteriorly with excellent results, especially for primary hernias [9, 10]. Moreover, this distrust of the minimally invasive approach was initially fueled by the risk of significant complications: visceral lesions during TAPP and vascular lesions during TEP [1, 3]. However, as of today, the International Guidelines published by the Hernia Surge Group have demonstrated the safety of the laparoendoscopic approach for inguinal hernias, especially concerning complications, and the results in terms of postoperative pain and recurrences are substantially comparable to groin hernia repair performed anteriorly [1]. From the analysis of the data, we are unable to trace the specific types of complications. However, we can see how they have progressively decreased and how mortality after 30 days is in line with the guidelines. The low conversion rate and complications could indicate that the centers performing TAPP or TEP are medium–high volume centers [12, 13]. In our study, we have no breakdown of the types of approach as the evaluation code does not provide differentiation in the kind of approach; however, we are aware that in Europe, transperitoneal operations are less prevalent compared to preperitoneal; while in Germany, according to the data of the German Hernia Surge Register, more TAPPs are performed, and only 20% are TEP; in Sweden and Switzerland, the preperitoneal approach is preferred [14, 15].

In addition to the technical difficulties and a long learning curve, in Italy, a further obstacle to the spread of the laparoscopic technique has been the remuneration of the operation that, regardless of whether the hernia was monolateral, bilateral or recurrent, is remunerated in the same way as a monolateral open hernioplasty. Although some recent studies have reported advantages in healthcare expenditure for laparoendoscopic procedures, this figure is probably distorted by the type of healthcare system adopted in each country [16, 17]. In some countries, the healthcare systems are welfarist, while in others, they are purely insurance-based, and in others still, they are mixed, so the impact of the reimbursement system could affect the push for health insurance in different ways. Moreover, as happened in Italy, the coding system has not been steadily updated, which has led to a lack of alignment with minimally invasive procedures. In Italy, only appendicectomy and cholecystectomy have a specific code when performed laparoscopically. At the same time, the other operations are associated with the laparoscopy code, yet the DRG (Diagnosis Related Group) reimbursement does not change. Reimbursement increases when a second procedure, such as adhesiolysis, is associated with the primary procedure, even if the lysis was performed on a single adhesion that would not have affected the hernioplasty approach. A recent paper by Aydin et al. showed that the cost of an anterior approach is similar to TAPP. However, the costs of hospital stay and anesthesia for each type of procedure are not reported, and bilateral and recurrence are compared [17]. The preperitoneal and transperitoneal approach does not seem to be related to a difference in expenditure. However, suppose the results of TEP and TAPP, as highlighted by the Guidelines, are equivalent, it is unthinkable that the difference in cost is determined only by the cost of the suture to close the peritoneum [1, 16, 17]. Other factors that could affect costs are complications. However, visceral and vascular lesions, in particular, have a very low incidence, so it is difficult to evaluate how much they affect costs [1, 4]. Unlike anterior approaches, high-energy devices (HED) could affect the cost of operations performed with the laparoendoscopic approach. However, as reported by Botteri et al. in a recent survey, the use of HED in abdominal wall surgery is not frequent [18, 19].

Another observation could be made regarding materials used for hernia repair. This information was not evaluable from the available dataset. Still, since there is a growing interest in using alternative materials for mesh, it could be interesting to make a further evaluation on the impact of costs on outcomes of their implementation into clinical practice. [20, 21]

Finally, as reported by Bracale et al., one of the major limitations in comparing laparoendoscopic and open surgical techniques is that scientific papers often compare bilateral vs monolateral hernias. [9]

From the data analysis, we can observe that there has been a progressive increase in the laparoendoscopic approach to inguinal hernia repair in Italy, together with an increase in the number of emergency operations performed for incarcerated hernias, showing a boost in confidence in the minimally invasive approach to inguinal hernioplasty even in more complex situations. In the literature, there are currently single experiences of some centers that demonstrate the operation's feasibility in safety, but with limits to the approach, regardless of the type of laparoendoscopic technique [9]; the guidelines of the Hernia Surge Group have not recommended the laparoscopic approach, but the advice is to select the method on a case-by-case basis [1].

It would have been interesting to have a better definition of the associated comorbidities to observe whether complications and mortality increased in correlation with some of them, as reported by some studies and guidelines [1]. However, from the analysis of the discharge codes, it was impossible to obtain reliable data on complications, likely because, due to the retrospective design of the registry, there was a lack of focus by the compilers.

## Conclusions

The findings of the present study have shown a first snapshot of the use of minimally invasive techniques for groin hernias in Italy, with substantial compliance with the international guidelines. The most relevant observation that could be made according to our analysis was that the adoption of the laparoscopic approach knew a slow but steady increase from 2015 onward. Undoubtedly, improving the attention paid by medical staff to coding is indispensable, together with a revision of remuneration values, especially in universal-coverage healthcare systems.

## Supplementary Information

Below is the link to the electronic supplementary material.

Supplementary file1 (DOCX 19 KB)

Supplementary file2 (DOCX 929 KB)

Supplementary file3 (DOCX 916 KB)

## References

[1] International guidelines for groin hernia management (2018). HerniaSurge Group. Hernia.

[2] Miserez M, Peeters E, Aufenacker T, Bouillot JL, Campanelli G, Conze J, Fortelny R, Heikkinen T, Jorgensen LN, Kukleta J, Morales-Conde S, Nordin P, Schumpelick V, Smedberg S, Smietanski M, Weber G, Simons MP (2014). Update with level 1 studies of the European Hernia Society guidelines on the treatment of inguinal hernia in adult patients. Hernia.

[3] Sartori A, De Luca M, Noaro G, Piatto G, Pignata G, Di Leo A, Lauro E, Andreuccetti JJ (2021). Rare Intraoperative and Postoperative Complications After Transabdominal Laparoscopic Hernia Repair: Results from the Multicenter Wall Hernia Group Registry. Laparoendosc Adv Surg Tech A.

[4] Kingsnoth A, LeBlanc K (2003). Hernias: Inguinal and Incisional. Lancet.

[5] Pisanu A, Podda M, Saba A, Porceddu G, Uccheddu A (2015). Meta-analysis and review of prospective randomized trials comparing laparoscopic and Lichtenstein techniques in recurrent inguinal hernia repair. Hernia.

[6] Deyo RA, Cherkin DC, Ciol MA (1992). Adapting a clinical comorbidity index for use with ICD-9-CM administrative databases. J Clin Epidemiol.

[7] Tran H, Tran K, Turingan I, Zajkowska M, Lam V, Hawthorne W (2015). Single-incision laparoscopic inguinal herniorraphy with telescopic extraperitoneal dissection: technical aspects and potential benefits. Hernia.

[8] Tschuor C, Metzger J, Clavien PA, Vonlanthen R, Lehmann K (2015). Inguinal hernia repair in Switzerland. Hernia.

[9] Arezzo A, Francis N, Mintz Y, Adamina M, Antoniou SA, Bouvy N, Copaescu C, de Manzini N, Di Lorenzo N, Morales-Conde S, Müller-Stich BP, Nickel F, Popa D, Tait D, Thomas C, Nimmo S, Paraskevis D, Pietrabissa A, EAES Group of Experts for Recovery Amid COVID-19 Pandemic (2021). EAES Recommendations for Recovery Plan in Minimally Invasive Surgery Amid COVID-19 Pandemic. Surg Endosc.

[10] Botteri E, Podda M, Sartori A (2020). The COVID-19 pandemic should not take us back to the prelaparoscopic era. J Trauma Acute Care Surg.

[11] Ayuso SA, Marturano MN, Katzen MM, Aladegbami BG, Augenstein VA (2022). Laparoscopic versus robotic inguinal hernia repair: a single-center case-matched study. Surg Endosc.

[12] Merola G, Cavallaro G, Iorio O, Frascio M, Pontecorvi E, Corcione F, Andreuccetti J, Pignata G, Stabilini C (2020). Bracale U Learning curve in open inguinal hernia repair: a quality improvement multicentre study about Lichtenstein technique. Hernia.

[13] Bracale U, Merola G, Sciuto A, Cavallaro G, Andreuccetti J, Pignata GJ (2019). Achieving the learning curve in laparoscopic inguinal hernia repair by tapp: a Quality Improvement Study. Invest Surg.

[14] Köckerling F, Lorenz R, Reinpold W, Zarras K, Conze J, Kuthe A, Lammers B, Stechemesser B, Mayer F, Fortelny R, Hoffmann H, Kukleta J, Weyhe D (2021). What is the reality in outpatient vs inpatient groin hernia repair? An analysis from the Herniamed Registry. Hernia.

[15] Gass M, Banz VM, Rosella L, Adamina M, Candinas D (2012). Gu¨ller U (2012) TAPP or TEP? Population-based analysis of prospective data on 4,552 patients undergoing endoscopic inguinal hernia repair. World J Surg.

[16] Mongelli F, Vajana AFT, FitzGerald M, Cafarotti S, Lucchelli M, Proietti F, Di Giuseppe M, La Regina DJ (2019). Open and Laparoscopic Inguinal Hernia Surgery: A Cost Analysis. Laparoendosc Adv Surg Tech A.

[17] Aydin M, Fikatas P, Denecke C, Pratschke J, Raakow J (2021). Cost analysis of inguinal hernia repair: the influence of clinical and hernia-specific factors. Hernia.

[18] Botteri E, Podda M, Arezzo A, Vettoretto N, Sartori A, Agrusa A, Allaix ME, Anania G, Brachet Contul R, Caracino V, Cassinotti E, Cuccurullo D, D'Ambrosio G, Milone M, Muttillo I, Petz WL, Pisano M, Guerrieri M, Silecchia G (2021). Agresta FCurrent status on the adoption of high energy devices in Italy: An Italian Society for Endoscopic Surgery and New Technologies (SICE) national survey. Surg Endosc.

[19] Shimpei O, Yuji K, Atsuyuki M, Yuichi T, Yasuyuki F, Shunsuke O (2017). Ultrasonic energy device versus monopolar energy device in laparoscopic transabdominal preperitoneal (TAPP) inguinal hernia repair. Updates Surg.

[20] Pizza F, D'Antonio D, Lucido FS, Del Rio P, Dell'Isola C, Brusciano L, Tolone S, Docimo L, Gambardella C (2022). Is absorbable mesh useful in preventing parastomal hernia after emergency surgery? The Parthenope study. Hernia.

[21] Pizza F, D'Antonio D, Ronchi A, Lucido FS, Brusciano L, Marvaso A, Dell'Isola C, Gambardella C (2021). Prophylactic sublay non-absorbable mesh positioning following midline laparotomy in a clean-contaminated field: randomized clinical trial (PROMETHEUS). Br J Surg.

