# Supplementary material for: Nationwide analysis of laparoscopic groin hernia repair in Italy from 2015 to 2020
Source: Updates Surg. 2022 Sep 7;75(1):77–84. doi: 10.1007/s13304-022-01374-7 (PMC9450816; doi:10.1007/s13304-022-01374-7)
Supplement: Supplementary file 1 — Supplementary file1 (DOCX 19 KB) [file 13304_2022_1374_MOESM1_ESM.docx]

| **Region** | **Year** | | | | | |
| --- | --- | --- | --- | --- | --- | --- |
|  | **2015** | **2016** | **2017** | **2018** | **2019** | **2020** |
| **Piemonte** | 4384226 | 4370348 | 4349911 | 4328565 | 4311217 | 4274945 |
| **Valle d'Aosta** | 127030 | 126677 | 126213 | 125653 | 125034 | 124089 |
| **Lombardia** | 9958447 | 9970419 | 9986962 | 10010833 | 10027602 | 9981554 |
| **PA di Trento** | 1059765 | 1063734 | 1068738 | 1074034 | 1078069 | 1077078 |
| **Veneto** | 4890648 | 4883373 | 4880936 | 4884590 | 4879133 | 4869830 |
| **Friuli Venezia Giulia** | 1216208 | 1212809 | 1211155 | 1210414 | 1206216 | 1201510 |
| **Liguria** | 1557742 | 1551379 | 1541541 | 1532980 | 1524826 | 1518495 |
| **Emilia-Romagna** | 4435480 | 4439768 | 4445920 | 4459453 | 4464119 | 4438937 |
| **Northern Italy** | 27629546 | 27618507 | 27611376 | 27626522 | 27616216 | 27486438 |
| **Toscana** | 3726422 | 3721391 | 3712048 | 3701343 | 3692555 | 3692865 |
| **Umbria** | 884092 | 880992 | 876477 | 873744 | 870165 | 865452 |
| **Marche** | 1538442 | 1532460 | 1526331 | 1520321 | 1512672 | 1498236 |
| **Lazio** | 5761508 | 5774092 | 5774606 | 5773076 | 5755700 | 5730399 |
| **Abruzzo** | 1319294 | 1313930 | 1306059 | 1300645 | 1293941 | 1281012 |
| **Central Italy** | 11910464 | 11908935 | 11889462 | 11868484 | 11831092 | 11786952 |
| **Molise** | 310026 | 308400 | 306564 | 303790 | 300516 | 294294 |
| **Campania** | 5790783 | 5776654 | 5762889 | 5740291 | 5712143 | 5624260 |
| **Puglia** | 4043735 | 4024067 | 4000966 | 3975528 | 3953305 | 3933777 |
| **Basilicata** | 569887 | 566405 | 562968 | 558587 | 553254 | 545130 |
| **Calabria** | 1943085 | 1935097 | 1924257 | 1912021 | 1894110 | 1860601 |
| **Southern italy** | 25887274 | 25833488 | 25753165 | 25659346 | 25538361 | 25326026 |
| **Sicilia** | 5004400 | 4977900 | 4942188 | 4908548 | 4875290 | 4833705 |
| **Sardegna** | 1642492 | 1636839 | 1631040 | 1622257 | 1611621 | 1590044 |
| **Island** | 6646892 | 6614739 | 6573228 | 6530805 | 6486911 | 6423749 |

***Supplemental Table 1*** Italian population on 31^st^ December from 2015 to 2020 the Italian National Institute of Statistics (ISTAT) [11]
