# Supplementary material for: Nationwide analysis of laparoscopic groin hernia repair in Italy from 2015 to 2020
Source: Updates Surg. 2022 Sep 7;75(1):77–84. doi: 10.1007/s13304-022-01374-7 (PMC9450816; doi:10.1007/s13304-022-01374-7)
Supplement: Supplementary file 2 — Supplementary file2 (DOCX 929 KB) [file 13304_2022_1374_MOESM2_ESM.docx]

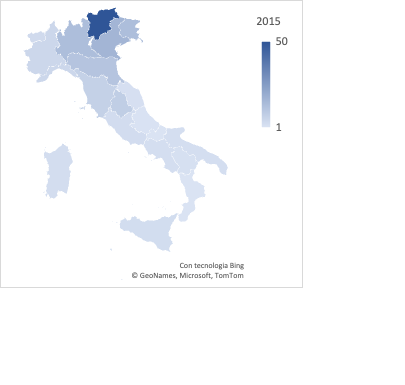

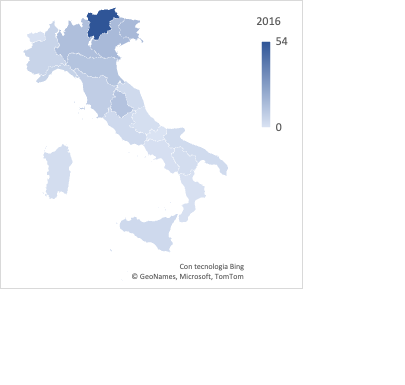


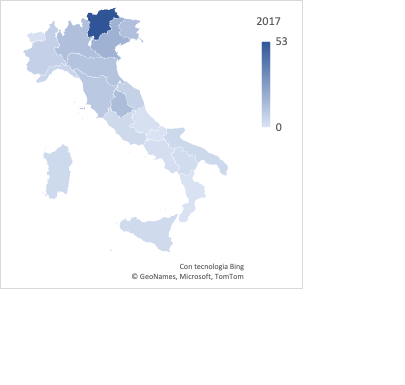

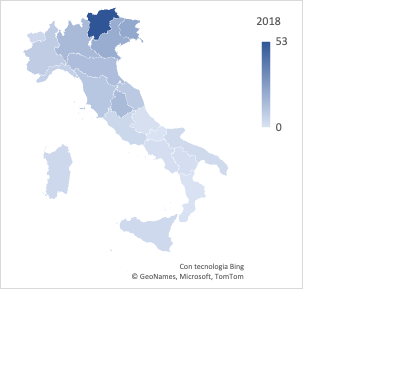


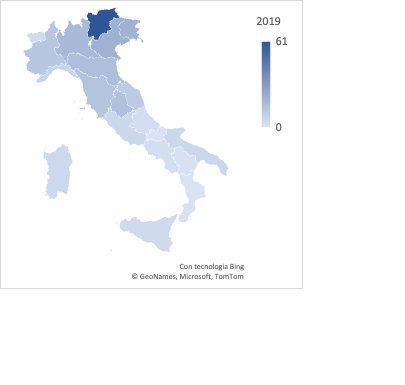

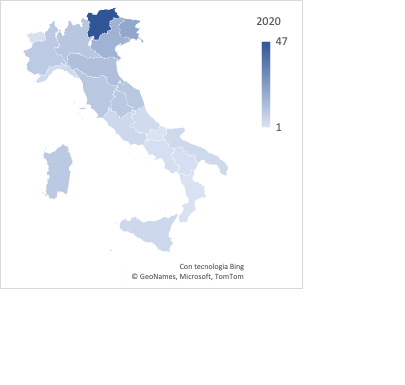


**Supplemental Figure 1** Annual Interventions Rate (AIR) for elective laparoscopic groin hernia procedures (100,000 inhabitants) in Italy from 2015 to 2020 (sources Agenas and Italian National Institute of Statistics (2022) Resident population on 31st December. ISTAT. <http://dati.istat> .it/?lang=en#.)
