# Supplementary material for: Nationwide analysis of laparoscopic groin hernia repair in Italy from 2015 to 2020
Source: Updates Surg. 2022 Sep 7;75(1):77–84. doi: 10.1007/s13304-022-01374-7 (PMC9450816; doi:10.1007/s13304-022-01374-7)
Supplement: Supplementary file 3 — Supplementary file3 (DOCX 916 KB) [file 13304_2022_1374_MOESM3_ESM.docx]

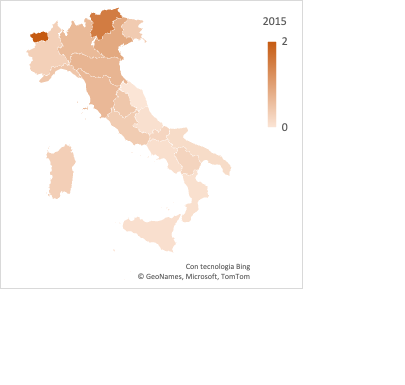

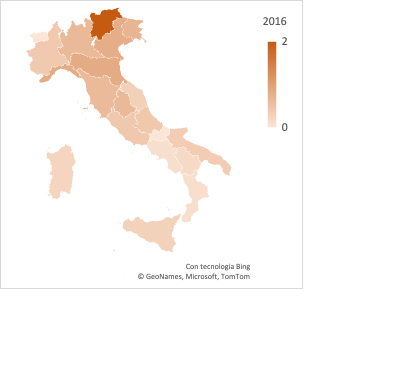


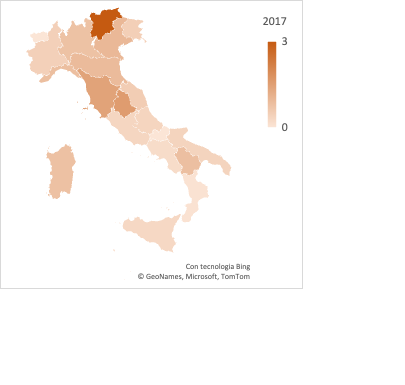

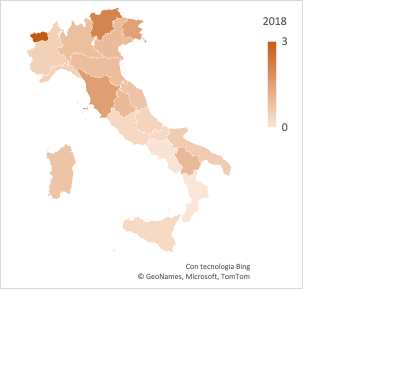


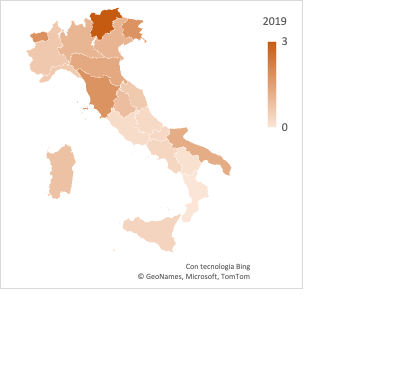

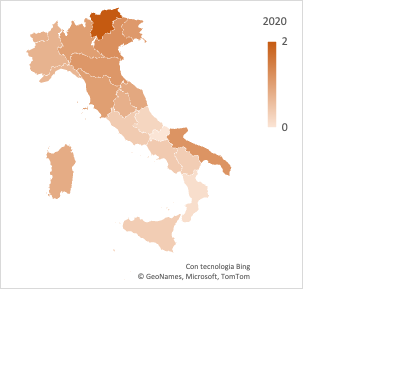


**Supplemental Figure 2.** Annual Interventions Rate (AIR) for urgent laparoscopic groin hernia procedures (100,000 inhabitants) in Italy from 2015 to 2020 (sources Agenas and Italian National Institute of Statistics (2022) Resident population on 31st December. ISTAT. <http://dati.istat> .it/?lang=en#.)
